# Particulate-free porous silicon networks for efficient capacitive deionization water desalination

**DOI:** 10.1038/srep24680

**Published:** 2016-04-22

**Authors:** Thomas Metke, Andrew S. Westover, Rachel Carter, Landon Oakes, Anna Douglas, Cary L. Pint

**Affiliations:** 1 Department of Mechanical Engineering, Vanderbilt University, Nashville, TN 37235, USA; 2 Interdisciplinary Materials Science Program, Vanderbilt University, Nashville, TN 37235, USA

## Abstract

Energy efficient water desalination processes employing low-cost and earth-abundant materials is a critical step to sustainably manage future human needs for clean water resources. Here we demonstrate that porous silicon – a material harnessing earth abundance, cost, and environmental/biological compatibility is a candidate material for water desalination. With appropriate surface passivation of the porous silicon material to prevent surface corrosion in aqueous environments, we show that porous silicon templates can enable salt removal in capacitive deionization (CDI) ranging from 0.36% by mass at the onset from fresh to brackish water (10 mM, or 0.06% salinity) to 0.52% in ocean water salt concentrations (500 mM, or ~0.3% salinity). This is on par with reports of most carbon nanomaterial based CDI systems based on particulate electrodes and covers the full salinity range required of a CDI system with a total ocean-to-fresh water required energy input of ~1.45 Wh/L. The use of porous silicon for CDI enables new routes to directly couple water desalination technology with microfluidic systems and photovoltaics that natively use silicon materials, while mitigating adverse effects of water contamination occurring from nanoparticulate-based CDI electrodes.

Water desalination is a key challenge facing human sustainability in coming decades^1^. Ninety-eight percent of all the water on earth is salt water, leaving only two percent of water usable for society^2^. Increasing population density and rapid consumption of fresh water resources will cause major water shortages in coming years from overuse and contamination^1^. Desalination methods can transform salt water reserves to usable freshwater sources, but require significant amounts of energy input to be successful^3^,^4^,^5^,^6^. Despite advances in desalination technology, the cost and scale of currently available routes for water desalination remain too expensive for use in developing countries, where water shortage is already a current problem^7^. Therefore, new techniques that can lead to low-cost materials and energy efficient processes for water desalination are of critical importance to a sustainable future.

Currently, the most commonly used methods of desalination include multi-stage flash distillation and reverse osmosis^4^,^5^,^6^. Flash distillation is the most energy expensive, requiring ~25 Wh/L of energy that is lost upon condensation^8^,^9^. Reverse osmosis (RO) is more energy efficient than flash distillation (5 Wh/L), but utilizes membranes most often made from cellulose or polyamides, and used to remove salt or contaminants under a very large applied pressure^8^,^9^,^10^,^11^. As a result, this requires costly centralized infrastructure that is not straight-forward to scale-down. Capacitive Deionization (CDI) has the potential to be more efficient than either of these techniques with a total energy consumption of as little as 1.1 Wh/L^9^,^12^, and promise for system development at small scales relevant to families or individuals. Capacitance deionization operates on the principle of a low-voltage (<2 V) electric bias applied across electrodes immersed in salt water to assemble the dissolved salt on the electrode surface^13^. The formation of this salt layer in the form of an electrochemical double layer on materials with ultra-high surface areas, such as many forms of carbons, can lead to the complete extraction of salt ions from water. Furthermore, when the electrodes are discharged to expel the ions back into another medium, a significant portion of the energy that is utilized for desalination can be recycled in the process, greatly improving the efficiency of capacitive deionization^14^. Recent efforts have most notably demonstrated materials such as metal organic porous carbon frameworks and three dimensional graphene architectures with measured capacitive desalination that gives promise to practical CDI technology^15^,^16^,^17^,^18^. At the current time however there are still several key challenges for CDI technologies including achieving high efficiency for water desalination, maintaining electrodes compatible with large-scale manufacturing, and controlling the electrode structure to inhibit fouling or deactivation^19^. High performance materials for these applications, such as single-walled carbon nanotubes or 3D graphene foams^17^, result in the best performance but rely on small-scale processing that remains expensive and research-centered, generate particulates that can contaminate the water source, and provide highly porous networks prone to fouling^20^,^21^. Activated carbon is the most cost-viable option for CDI electrodes, but bring many of the same challenges as other carbons for practical use and require expensive processing to cast particulates into robust templates resistant to salt water flow environments.

Here we demonstrate that porous silicon – a material exhibiting earth abundance^22^, low cost^23^, environmental/biological compatibility^24^,^25^ and readily adaptable into the already well developed Si manufacturing infrastructure^23^, as a candidate material for water desalination. Unlike the assembly of carbon-based particulates, porous silicon templates can be etched into bulk silicon using processes central to semiconductor manufacturing and dating back over 6 decades of material development and building on raw materials with cost as little as $2/kg^23^,^26^. Additionally the widespread use of Si has led to very well developed silicon processing infrastructure^23^ which is in contrast to carbon based devices where the necessary infrastructure for large scale processing has yet to be developed, even though carbon is an earth abundant and attractive material. The porosity, thickness, and feature size (surface area) can be varied over orders of magnitude^27^,^28^,^29^ enabling a level of control that can mitigate fouling, optimize durability under flow, and efficiently enable salt removal without the release of free particulates into the flow. As this material has promise to overcome many of the key problems facing current CDI technology, our results indicate salt removal performance up to 0.52% by mass with performance maintained across the whole range of brackish water conditions, which is comparable or better than other state-of-the-art carbon particulate-based electrodes. This not only gives promise to this material for CDI applications, but opens new routes for integration into systems such as microfluidics^30^ or solar cells^31^, that are synergistic with desalination technology and rooted in silicon processing architectures.

## Results

**Figure 1:**
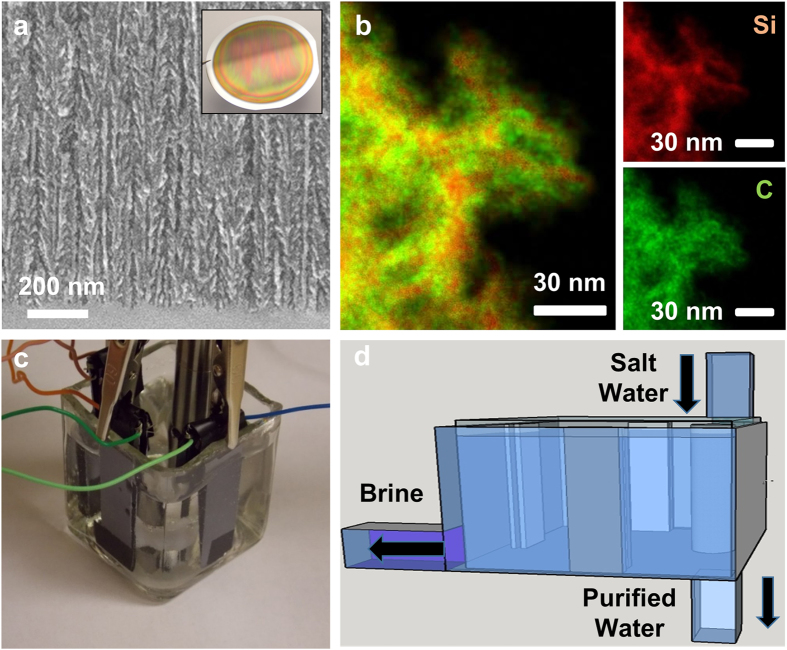
Porous silicon based CDI electrodes. (**a**) Cross-sectional SEM image of a carbon passivated porous silicon electrode for water desalination. (**b**) Analytical elemental TEM analysis a carbon passivated porous silicon material showing uniform carbon passivation on the material. (**c**) Photograph of the CDI testing cell combining 4 pairs of silicon-based electrodes, and (**d**) illustration of a CDI module in the context of the testing apparatus for continuous flow-through CDI water desalination using porous silicon.

Porous silicon was produced by electrochemically etching full 4” Si wafers in an AMMT commercial etching system to create a porous silicon material with ~75% porosity and depth that varies with etch time. Whereas porous silicon is natively poorly suited for operation in aqueous environments, surface passivation of the porous silicon material with carbon^32^,^33^,^34^,^35^ was used to effectively transform the highly porous material into an electrochemically stable template for water desalination. The ability to fine tune the porous Si structure allows for optimization of the desalination performance and antifouling capability, and the carbon coating presents an electrode interface capturing the properties of carbon that lead to highly efficient salt electrosorption. Electron microscopy analysis shows representative images confirming both the porous network that is integrated onto the silicon wafer (Fig. 1a) as well as a nanostructured material that is conformally passivated with carbon (Fig. 1b). Unlike other routes to produce CDI electrodes, this material has a pore architecture controlled by the silicon electrochemical etching process, a structure that is tethered to a solid surface to inhibit particulate removal, and a stable interface for salt removal that is mediated by the carbon passivation layer. This gives a level of controllability in electrode design that can be modulated based on the electrochemical etching process, leading to routes to produce integrated materials that can overcome challenges of fouling and flow-resistance critical for CDI electrode systems.

In order to generate measureable changes in the salinity of aqueous solutions, a test cell was designed that involves four parallel sets of electrode pairs assigned to each face of a square container filled with salt water with a circulator in the center to maintain an equilibrium salinity of the water during experiments. (Figure 1c) This design is envisioned to be a modular component of an engineered CDI desalination cell that can sequentially purify salt water until a tolerable fresh water concentration is achieved. (Figure 1d) In the flow cell employed for this study, the electrodes were separated by ~1.1 mm to allow ample water flow between the electrodes and a conductivity meter was utilized to assess the change in salinity of the aqueous solution in the cell. Although a full process scale design for continuous desalination is beyond the present study, it is anticipated this could be carried out using two routes at two different scales. First, a modular CDI cell composed of a series of cells similar to that illustrated in Fig. 1d would involve a sequence where salt water and brine are sequentially cycled through the modular series of cells to lead to fresh water and concentrated brine. An alternative to this that would be effective at small scales is the incorporation of porous silicon into microfluidic flow channels whose channel dimensions and flow rate would be optimized for salt electrosorption, and salt water and brine could similarly be repetitively cycled. Notably the efficient recovery of the brine in both cases can lead to the recovery of the majority of the initial energy input into the system.

**Figure 2:**
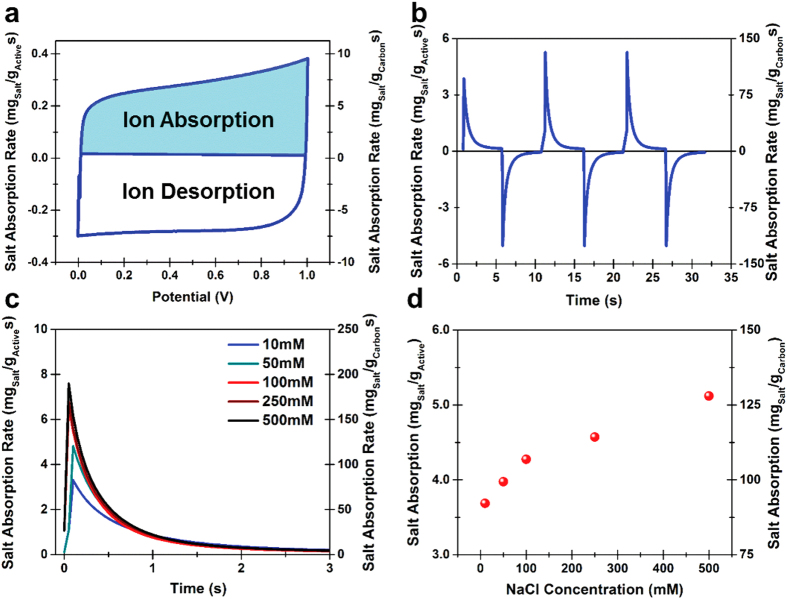
Porous silicon salt removal in different salt-water concentrations. (**a**) Cyclic voltammetry analysis at 100 mV/s scan rate in 500 mM NaCl salt water solution, with labeled salt absorption and desorption segments. (**b**). Current profile for 3 cycles of potentiostatic desalination cycling in 500 mM NaCl solutions with porous silicon CDI electrodes, (**c**) Potentiostatic salt removal profiles for NaCl solutions ranging from 10 mM (near-fresh) to 500 mM (near-ocean water concentration) NaCl. (**d**) Specific salt removal capacity as a function of water concentration based on results in part (**c**).

To first assess the ability of this material for salt removal, cyclic voltammetry (CV) measurements were performed in 500 mM NaCl. (Figure 2a) CV measurements confirm the stable electrochemical interface between salt water and the porous silicon material in the voltage range relevant for CDI experiments, between 0 V and 1 V. The total width of the box in the CV curve in Fig. 2a corresponds to the total charge associated with both salt absorption and salt desorption from the porous silicon electrode, appropriately labeled. In the CV curves it is apparent that the majority of the ions stored can be released from the electrodes effectively rejuvenating them to allow for continuous cycles and recovery of a significant portion of the energy input. To further characterize this system, and to understand the ability to both remove ions and then rejuvenate the electrodes we performed potentiostatic testing to porous silicon CDI electrodes immersed in salt water with varying NaCl concentration ranging from high concentrations relevant to salt concentrations in ocean water (500 mM) to lower concentrations representing moderately brackish water (10 mM). (Figure 2b,c) Salt removal is associated with a large spike in current that is due to the rapid assembly of salt ions onto the porous silicon surface, with a current tail that exponentially decays over time. This potentiostatic testing indicates that this passivated porous silicon material can yield salt removal at high salt concentrations of up to 5.2 mg NaCl per gram of porous silicon material (5.2 mg/g), and can still maintain salt removal of 3.8 mg/g at low salt concentrations that border the transition from brackish to fresh water (Fig. 2d). Importantly, these results are normalized to the combined mass of the porous silicon and the carbon interface, which forms the skeleton and passivating interface for this electrode system, respectively. Isolating only the performance of the active carbon material interface, our results indicate that ion removal capacity could be as high as ~125 mg/g_carbon_ if the optimal performance could be implemented in a flow electrode setup. Whereas such an ultrathin porous carbon material would likely not have the native mechanical integrity to be a practical self-supporting CDI flow electrode, the combined function of the carbon and porous silicon give promise to a tunable architecture to mitigate fouling, high electrosorption properties, and a material that can be seamlessly integrated with silicon-based applications.

In order to assess how these electrodes compare to RO and flash distillation we used the electrosorption results to calculate the net energy required by the system to desalinate ocean and brackish water. Coulombic efficiency of ~95–98% and an energy efficiency of ~85% was measured in our system. Based on these values, the net amount of energy it would take to desalinate salt water from either ocean water concentrations (35000 ppm) or brackish water concentrations (30000–5000 ppm) to fresh drinkable water (<500 ppm) was calculated. Our results indicate that it would take ~1.45 Wh/L to purify ocean water to fresh water and 0.25–1.25 Wh/L to purify brackish water to fresh water emphasizing the ability of these electrodes to provide a significant energy advantage over RO (>5 Wh/L) and Flash Distillation (~30 Wh/L)^8^.

**Figure 3:**
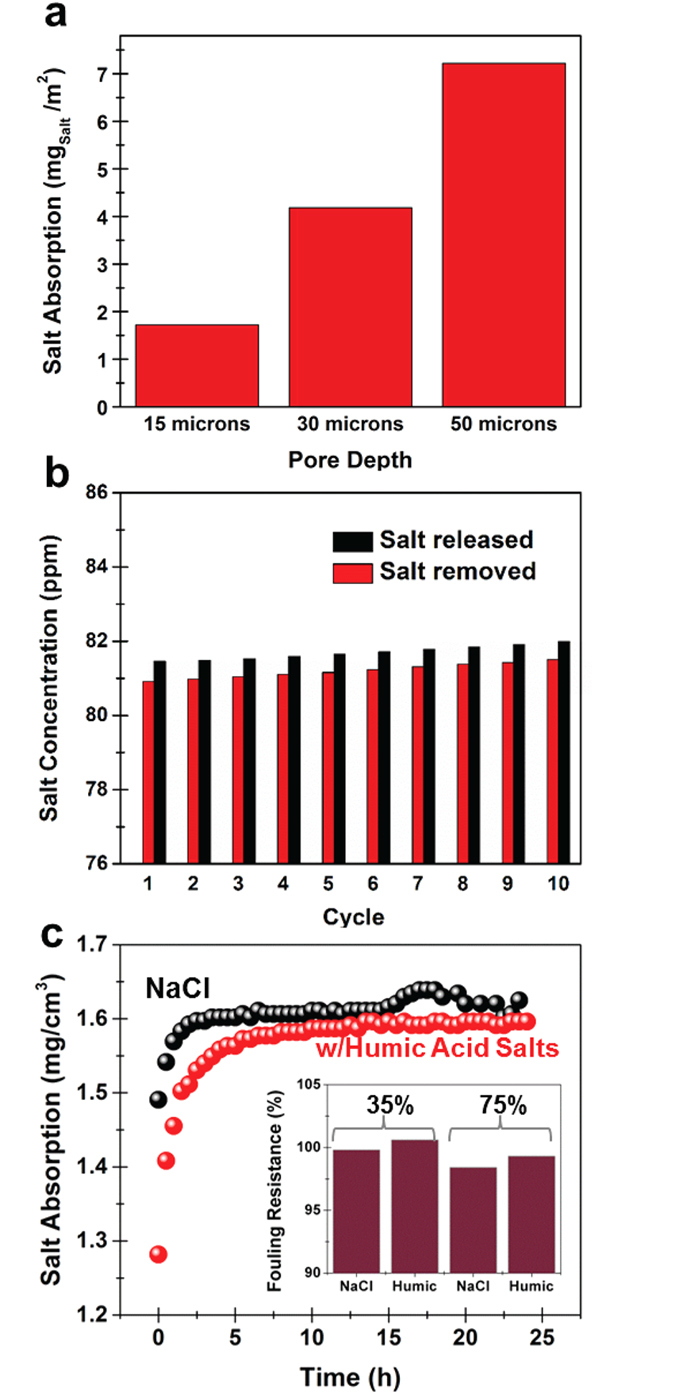
Effect of porous silicon depth on CDI salt removal capability. (**a**) Average salt removal capacity per CDI electrode area over 10 cycles based on porous silicon layers with thickness of ~15, 30, and 50 μm, respectively. (**b**) Salt removal cycling in low-concentration (fresh) water showing salt absorption (red) and salt desorption back into water (black). (**c**) Galvanostatic salt absorption/desorption curves in pure NaCl (black) and with humic acid salt fouling additives (red). Inset shows fouling resistance comparing porous silicon electrodes with 35% and 75% porosity with and without fouling agent in the latter 15 hours of steady-state CDI measurements, emphasizing the absence of fouling from this electrode geometry.

One unique feature of porous silicon materials is the ability to control the porous silicon thickness, the porosity and pore size based on the total duration of electrochemical etching and the etching current density. In order to show the advantages that come from this ability to fine-tune the porous Si depth and structure we first performed CDI experiments on porous Si electrodes of differing pore depths and showed the effect of pore depth on the overall salt removal capability of porous silicon CDI electrodes. The specific salt removal measured as a function of the footprint area of porous silicon material (Fig. 3a) exhibits a linear correlation with porous silicon depth, with depths ranging up to ~50 μm in thickness. The use of deeper pores allows for the removal of more salt for a single, planar electrode. This emphasizes the possibility to easily develop significant amounts of active material integrated into a single electrode and enables more CDI active material produced in any one given etch process, yielding a versatile approach to developing functional electrodes for CDI operation. Furthermore, the thickest porous silicon layer used as a CDI electrode (~50 μm) was subjected to subsequent salt extraction cycles to demonstrate its longevity for stable CDI performance (Fig. 3b). The slight increasing baseline in Fig. 3b is associated with water evaporation in a module open to ambient conditions. Notably, whereas these results were obtained using low-concentration salt water due to the ease of resolving the total salt removal, this trend is consistent across the whole range of brackish water concentrations relevant for a CDI system.

One additional advantage that comes from the ability to fine tune the porosity of the porous Si is the potential to design a pore structure that can mitigate the effect of fouling. Figure 3c and inset shows the fouling performance of the porous Si CDI electrodes with 75% porosity and a comparison of 75% porosity and 35% porosity (inset). In order to simulate the effect of fouling we compared the long term cycling performance of the porous Si electrodes in the pure NaCl solution (1000 ppm-1 g/L) and compared that to the performance with an additional 3 ppm of humic acid salts^36^. Despite slightly different response of the electrodes in the first few hours of cycling, the stable long-term performance over a 24 hour period reaches a steady state performance that is relatively invariant with regard to the fouling agent. This implies that due to the ideal structure of porous silicon combining deep pore channels and pockets of electrosorption sites along the channels (Fig. 1a), fouling does not appear to present a significant challenge. This is further emphasized by comparing the fouling resistance, or the modification of the steady-state electro-sorption/desorption properties, in the latter (steady-state) 15 hours of cycling at two different porosities of 75% and 35% as inset in Fig. 3c that does not indicate a significant effect of fouling in these electrodes.

## Discussion

The results presented in Figs 1, 2, 3 support the principle that porous silicon networks are practical scaffolds for CDI applications over a wide range of practical salt concentrations ranging from ocean water to fresh water. Based on the accepted standards of the United States Office of Naval Research (ONR) and the Groundwater Foundation (GF), fresh water contains salt concentrations <500 ppm (salinity <0.05%) and brackish water involves concentrations ranging from 500–30,000 ppm (0.05–3% salinity). Ocean water has salinity near ~3.5%. In this spirit, the tests in Figs 2 and 3 range from saline water with salt concentrations near that of ocean water (~2.9% salinity, 500 mM) to near-fresh water concentrations (~0.06% salinity, 10 mM). The performance of porous silicon based CDI electrodes over this range of concentrations supports the principle of a multi-cell CDI system based on porous silicon materials that can maintain high efficiency in converting ocean water to fresh water.

**Figure 4:**
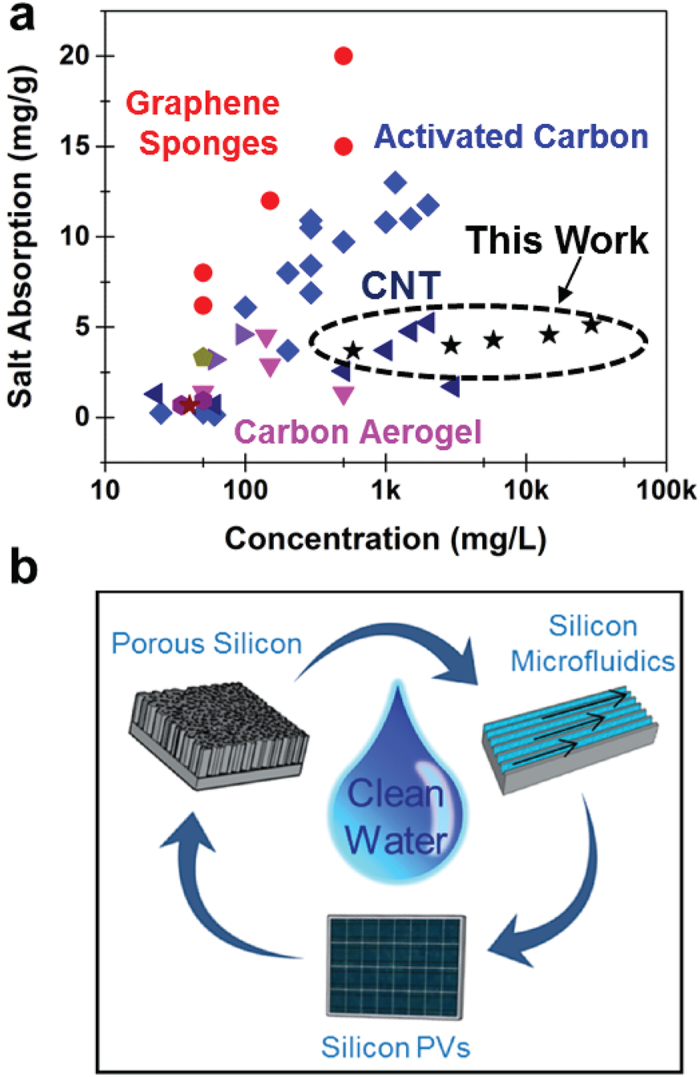
High performance of porous silicon CDI electrodes and integration vision. (**a**) Comparison of average specific salt removal capacity for porous silicon electrodes compared to other notable forms of carbon, such as 3D graphene sponges, carbon aerogels (CA), activated carbons (AC), and carbon nanotubes (CNTs)^17^,^37^,^38^. (**b**) Scheme emphasizing the vision of porous silicon integration with microfluidics and/or solar cells for technological water desalination platforms.

To further highlight the performance measured for porous silicon CDI electrodes, we compare the performance measured in this study against that reported for CDI electrodes produced with different carbon electrode materials^17^,^37^,^38^. (Figure 4a) To compare to these materials we report the average salt removal measured across the entire range of concentrations. This compares the integrated carbon coated porous silicon CDI electrodes against 3D Graphene foams, carbon aerogels (CA), activated carbon (AC), carbon nanotubes (CNTs), carbon nanofibers (CNFs), mesoporous carbons (MC) and other carbon based CDI electrodes. Notably, the majority of these materials involve nanoscale carbon materials with either nano- or micro-particulates that are pressed into a CDI electrode. The results we report are comparable to salt removal capacity of many of these state-of-the-art carbon materials and better than the general range of salt removal capacities of these different materials. Although our measured performance still lags behind the highest performing graphene-based electrode assemblies, our results indicate that routes to isolate mechanically robust free-form carbon electrodes from the porous silicon networks can potentially achieve such high performance. However, in contrast to stand-alone carbon CDI electrodes, the use of porous silicon templates for CDI opens up routes to directly couple CDI active materials into silicon platforms relevant to microfluidic flow systems or even such flow systems integrated on the backside of silicon photovoltaic cells. (Figure 4b) Recent efforts have emphasized the capability to etch porous silicon into the back-side of unused silicon in photovoltaic cells to enable the dual function of energy storage and energy conversion in a silicon solar cell^31^. The coupling of CDI technology into silicon can lead to even the more exciting prospect of integrating solar and desalination systems into directly coupled systems that, in contrast to reverse osmosis systems that require large-footprint infrastructure for operation, could be powered by cheap solar cells and operated at small scales in low-income families or communities for water purification.

In summary, building from the 2^nd^ most abundant element on earth, leveraging scalable processing routes, and producing controllable materials not involving free-formed particulates, as in other techniques, we show specific salt removal ranging from 3.8 mg/g to 5.2 mg/g ranging from fresh to ocean water salt concentrations, requiring energy input as little as 1.45 Wh/L to convert ocean water to drinking water. This is comparable or better in many cases to particulate carbon-based electrodes, with a tunable pore morphology and structure that can optimize CDI performance in different flow architectures and mitigate the effect of fouling. Unlike carbon-based particulate materials, this route leads to straightforward directions to seamlessly integrate CDI materials into silicon-based microfluidics or photovoltaic cells. This inspires a vision of cheap, local water desalination platforms powered by silicon photovoltaics that can be employed in low-income or developing countries without the necessity of external power input or high cost infrastructure.

## Methods

### Porous Silicon Fabrication

Porous silicon etching was performed in a commercial AMMT electrochemical system using full 4” boron-doped silicon wafers. The etch process was carried out using a 3:8 v/v HF (50% H_2_O by volume) and ethanol etch solution, and durations of 540 s, 1080 s, and 1800 s to produce different depths. Based on previous analysis, this technique yields a porous silicon material with ~75% porosity^32^,^34^. To modify the porosity to 35%, the etch current was modified to 10 mA/cm^2^. Following the P-Si etch process, the samples were washed in ethanol and stored in an Ar glove box until gas phase carbonization.

### Porous Silicon Surface Passivation

Carbon coatings were applied to the porous silicon materials in order to both prevent oxidation of the silicon in aqueous environments and reverse the effect of trap states at the surface. Porous silicon materials were loaded into a 1” Lindberg-Blue tube furnace and placed in the center the tube furnace. The sample was then flushed with Ar (1 slm) and H_2_ (200 sccm) during heating up to 650 °C. At a temperature of 650 °C, 10 sccm of C_2_H_2_ were included in the gas mixture, and the temperature was sequentially ramped to 750 °C and 850 °C over durations of 10 minutes in both cases. This route has been observed to produce conformal coatings of carbon that retain the structure of the porous silicon and yield significant corrosion resistance of the silicon in even the most aggressive electrochemical environments^32^,^39^. Material analysis of this surface passivated porous silicon was performed using scanning electron microscopy (Zeiss, Merlin) and transmission electron microscopy (FEI Osiris). Carbon mass was determined by weighing the porous Si before and after carbonization using a semi-microbalance.

### Capacitive Deionization Testing

Capacitive deionization testing was performed using two separate routes. The first route involves testing of electrodes in a two-electrode configuration using an MTI split flat cell. The second route involves the development of a cell involving four pairs of CDI electrodes in a square configuration that was continuously stirred or circulated for the duration of experiments (Fig. 1c,d). In the latter case, the total salt removal from the water was characterized based on a conductivity meter (Thermo Scientific, Orion Star A212) that was inserted into the cell following potentiostatic salt removal. For cyclic voltammetry tests, which were primarily tested in a two-electrode configuration, a voltage range of 0–1 V was utilized to inhibit hydrolysis of water. For potentiostatic tests, which were primarily carried out in the CDI cell, the current was monitored as a 1 V potential is applied to the cell (salt removal). In all cases, electrochemical tests were performed using a Metrohm autolab multichannel workstation. The energy analysis was performed based on galvanostatic charge discharge curves used for the cycling analysis in Fig. 3c, where the total charge absorbed was measured with the current and time and then translated into grams of NaCl, and the energy was calculated by integrating the charge and discharge curves respectively and multiplying by the charging current according to the following equation. *E* = ∫*IVdt*. The effect of fouling was determined by galvanostatically cycling the electrodes in an NaCl solution (1000 ppm) and similarly NaCl solution (1000 ppm) with the addition of 3 ppm of humic acid salts (Sigma Aldrich) similar to the procedure used by M. Mossad *et al*.^36^ The fouling resistance used for the inset in Fig. 3c. was calculated by comparing the performance of the Porous Si electrodes of 35 and 75% porosities after a steady state absorption capacity was reached with the performance after the full 24 hours of testing.

## Additional Information

**How to cite this article**: Metke, T. *et al*. Particulate-free porous silicon networks for efficient capacitive deionization water desalination. *Sci. Rep.* **6**, 24680; doi: 10.1038/srep24680 (2016).
